# Fumitremorgin C alleviates advanced glycation end products (AGE)-induced chondrocyte inflammation and collagen II and aggrecan degradation through sirtuin-1 (SIRT1)/nuclear factor (NF)-κB/ mitogen-activated protein kinase (MAPK)

**DOI:** 10.1080/21655979.2021.2024387

**Published:** 2022-02-02

**Authors:** Yeli Zhou, Jing Li, Chenglong Wang, Zheer Pan

**Affiliations:** Department of Orthopedics, The First Affiliated Hospital of Wenzhou Medical University, Wenzhou, China

**Keywords:** Fumitremorgin C, osteoarthritis, SIRT1, NF-kB, MAPK

## Abstract

Fumitremorgin C is a potent and selective inhibitor of the breast cancer resistance protein. This study aimed to explore the role of fumitremorgin C in osteoarthritis (OA) and disclose the underlying mechanism. The cell viability of AGE-treated SW1353 cells in the presence of fumitremorgin C was detected by Cell Counting Kit-8 (CCK-8) assay. The inflammation and extracellular matrix (ECM) deposition of AGE-induced SW1353 cells was respectively measured by enzyme linked immunosorbent assay (ELISA), immunofluorescence, and Western blot. The expression of SIRT1 and NF-KB/MAPK signal was examined by Western blot. After that, SIRT1 inhibitor EX527 was added to observe the mechanism of action of fumitremorgin C. Fumitremorgin C restored the cell viability of SW1353 cells injured by AGE. Furthermore, it alleviated inflammation and ECM degradation in AGE-induced SW1353 cell. The SIRT1 expression decreased by AGE was recovered upon fumitremorgin C to SW1353 cells. The ratio of phosphorylated p65 (p-p65) and p65, phosphorylated JNK (p-JNK) and JNK, and phosphorylated 38 (p-38) and 38 were increased by AGE treatment, which was recovered by fumitremorgin C addition. SIRT1 inhibitor EX527 reverts the repressive effects of fumitremorgin C on inflammation and ECM degradation in AGE-induced SW1353 cell. In conclusion, fumitremorgin C alleviates AGE-induced inflammation and the degradation of collagen II and aggrecan through SIRT1/NF-κB/MAPK, which reveals the underlying mechanism by which fumitremorgin C alleviates OA.

## Graphical abstract

**Figure:**
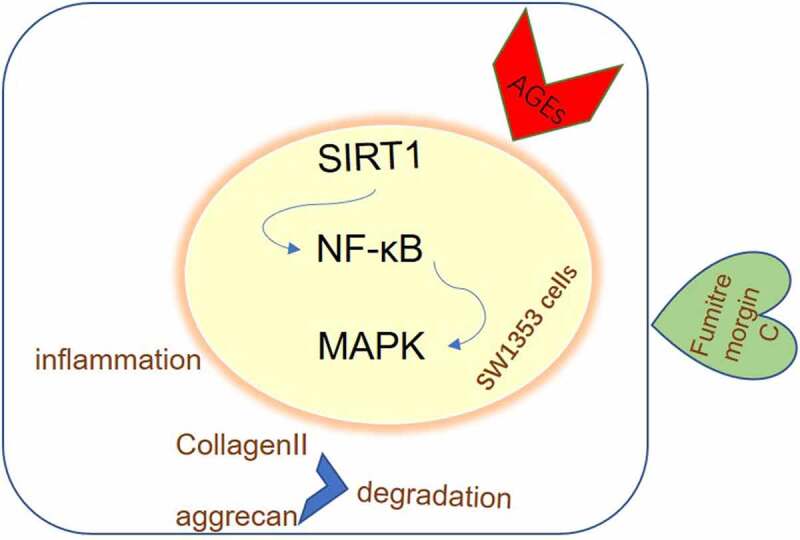

## Introduction

Osteoarthritis (OA) is a degenerative joint disease manifested as cartilage calcification, bone sclerosis, and persistent synovial inflammation in the joint [1,2]. This is one of the most prevalent disease in the elderly who are over 60 and symptoms including pain, stiffness, swelling, and tenderness have gradually disintegrated the happiness on patients’ faces [3]. Aging has been considered as one of the important risk factors of OA. In the process of aging, the non-enzymatic glycosylation of macromolecules leads to the production of AGEs, and the poor degradation of these products leads to the accumulation of a large number of age products in the joints, resulting in the imbalance of decomposition and metabolism of extracellular matrix. AGEs can induce the phosphorylation of NF-KB and MAPK family, including JNK and p38MAPK, and play a role in promoting collagen degradation [4,5]. Therefore, targeted inhibition of NF-KB/MAPK phosphorylation plays an important role in inhibiting age induced chondrocyte inflammation and extracellular matrix degradation.

SIRT1 has crucial anti-inflammatory and anti-cancer effects, and can modulate NF-KB and MAPK pathway [6–8], which is reported to be involved in the pathogenesis of OA [9]. SIRT1 is reported to be of great value in aging and age-related diseases [10]. Some studies hold that SIRT1 can expedite the deterioration and degradation of collagen II in arthritis mice [11]. Therefore, activating SIRT1 and inhibiting NF-KB/MAPK also play an important role in arthritis.

Fumitremorgin C is a potent and selective inhibitor of the breast cancer resistance protein [12] (BCRP/ABCG2). The latest research on fumitremorgin C indicates that it can inhibit the formation of RANKL-induced osteoclasts, inhibit the expression of NF-κB/MAPK signal protein and reduce inflammatory release [13]. Therefore, this paper presumes that fumitremorgin C can suppress the inflammation of AGE-induced chondrocytes and the degradation of collagen II and aggrecan by SIRT1/NF-KB/MAPK signaling pathway. The objective of this study is to reveal the effects of fumitremorgin C on proinflammatory factors release and the degradation of CollagenII and aggrecan, thereby providing evidence for the potential benefits of fumitremorgin C in the treatment of OA.

## Materials and methods

### Cell culture and reagents

The SW1353 chondrocytes was purchased from the American Type Culture Collection (catalog o. HTB 94; lot No. 63,591,988; passage number 22). They were cultured in RPMI 1640 medium (Life Technologies, Inc., Gaithersburg, MD) supplemented with 10% fetal bovine serum (FBS) (Life Technologies), 4 mM l-glutamine, and 1% penicillin–streptomycin antibiotic solution at 37°C, under 5% CO_2_. Fumitremorgin C, purity>98%, was provided from Shanghai Taiyuan Biological Technology Co., LTD and dissolved in dimethyl sulfoxide (DMSO) for the following use. AGE-BSA was purchased from Biovision (#2221, purity >98%) and used to stimulate the SW1353 for 24 h [5,14].

### CCK-8 assay

The chondrocyte suspension at the density of 3 × 10^4^ cells per well was plated into a 96-well plate and cultured for 24 h. Then, the chondrocytes were exposed to fumitremorgin C (0, 2.5, 5, and 10 μM/L). The viability of SW1353 cells was assessed after 24 h. Each well of the plate was added with 10 μL CCK-8 solution and incubated for 4 h at 37°C. The optical density value at 450 nm was detected and read with a microplate reader.

### ELISA

After corresponding treatments, the SW1353 chondrocyte supernatants were obtained for ELISA assays. The supernatant was collected after centrifugation at 500 g for 5 min. The levels of tumor necrosis factor (TNF)-α, interleukin (IL)-1β, and IL-6 in the supernatant were quantified using sandwich-ELISA kits from Bioassay Technology Laboratory (Shanghai, China) according to the manufacturer’s protocol. Absorbance at 450 nm was read on a microplate reader (Molecular Devices).

### Immunofluorescence assay

SW1353 cells were seeded at a density of 5 × 10^3^ per well in 24-well plates. Cells were washed by PBS for three times and fixed with 4% paraformaldehyde for 20 minutes at room temperature. The cells were then incubated with blocking solution (0.2% powdered milk, 1% BSA, and 0.01% Triton X-100). After incubation for 30 min at room temperature, the cells were treated with anti-collagen II (Invitrogen, 1:5,00), Aggrecan (1:500, Santa Cruz Biotechnology, Inc.) overnight at 4°C. Cells were washed with PBS and staining with secondary antibody (1:400, Santa Cruz Biotechnology, Inc.) for 1 h at 37°C. To visualize the nuclei, 4ʹ, 6-diamidino-2-phenylindole (DAPI, Sigma) was added for 10 min, and the cells were observed under a fluorescence microscope (Zeiss).

### Western blot

Chondrocytes with the indicated treatments were lysed by using RIPA lysis buffer (Beyotime Biotechnology, Jiangsu, China) to collect total protein. Protein concentration was determined using bicinchoninic acid (BCA) method. An equal amount (25 μg) of protein was separated by SDS-polyacrylamide gel electrophoresis and electrotransferred onto polyvinylidene fluoride membrane (Millipore, Billerica, MA, USA). The membranes were soaked with 5% skim milk at room temperature for 1 h and probed with specific primary antibodies (MMP3, 1:2000; MMP13, 1:1000; ADAMTS-5, 1:250; SIRT1, 1:1000; p-NF-κB p65, 1:1000; NF-κB P65, 1:1000; JNK, 1:2000; GAPDH, 1:5000. Abcam, England. aggrecan, 1:1000; ADAMTS-4, 1:1000; p38, 1:1000; p-JNK, 1:1000; p-p38, 1:1000; Santa Cruz Biotechnology, Inc.) were used for incubation at 4°C overnight. The membranes were then incubated with horseradish peroxidase-conjugated secondary antibody (1:10,000, Abcam, England) for 1 h at 37°C. Bands were visualized using enhanced chemiluminescence detection reagents (Pierce, Rockford, IL, USA). The intensity of the bands on the membranes was analyzed using ImageJ software.

### Statistical analyses

All statistical analyses were conducted with GraphPad Prism software (7.0; GraphPad software, San Diego, Calif). The results are shown as mean ± standard deviation of the mean and analyzed by one-way analysis of variance and Tukey’s post-hoc test.

## Results

### Fumitremorgin C elevates the cell viability of AGE-induced SW1353 cells

**Figure 1.**
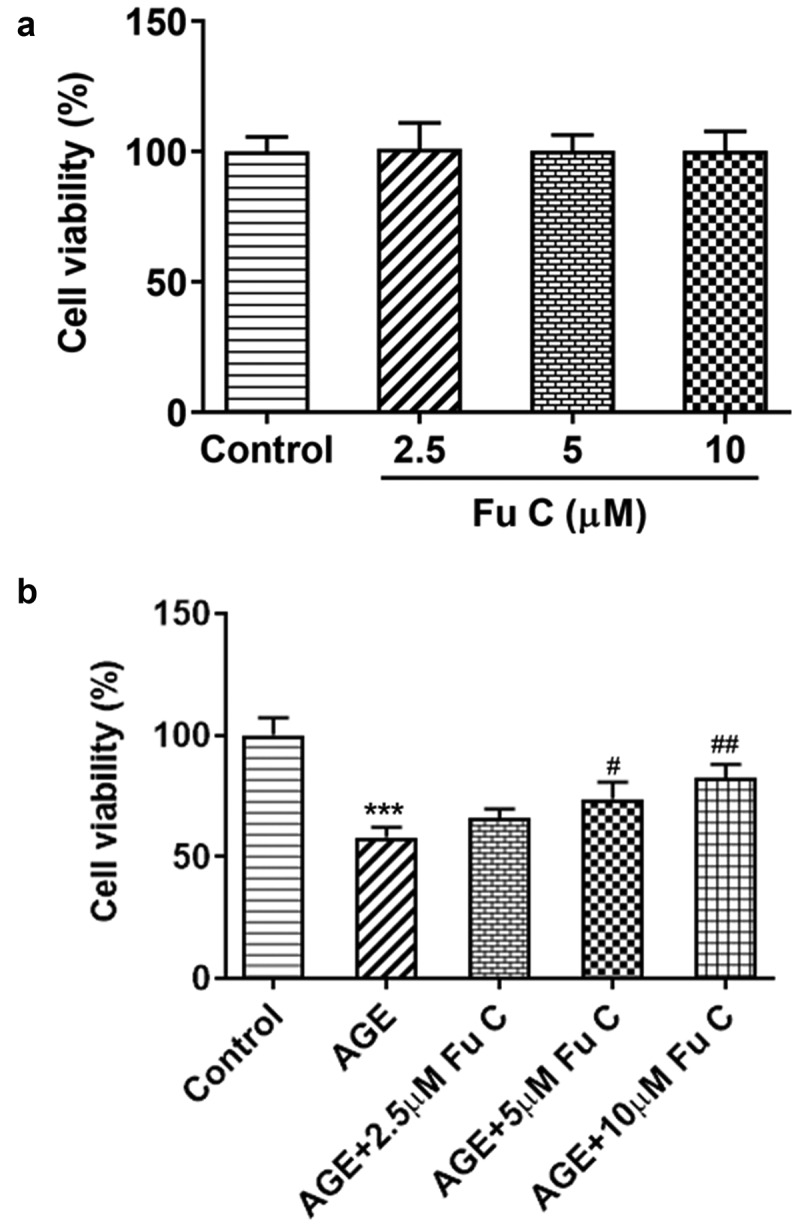
Fumitremorgin C elevates the cell viability of AGE-induced SW1353 cells. (a) The cell viability of SW1353 cells exposed to fumitremorgin C. (b) The cell viability of AGE-induced SW1353 cells exposed to fumitremorgin C. ***P < 0.001 Versus Control. ^#^P < 0.05, ^##^P < 0.01 Versus AGE. Fu C: Fumitremorgin C.

To detect the effects of fumitremorgin C on the cell abilities of AGE-induced SW1353 cells, the cell viability of SW1353 cells exposed to fumitremorgin C was measured. As presented in Figure 1(a), no significant change was found in SW1353 cells when doses of fumitremorgin C were added into the cells, confirming the nontoxicity of fumitremorgin C to the cells. As previous study indicated the 24 h of AGE incubation at the dose of 100 µg/ml did suppress the cell viability at a moderate extent, we chose this dose for the subsequent assays. Notably, fumitremorgin C (5, 10 µM) pretreatment for 2 h significantly restored the cell viability of SW1353 cells injured by AGE (Figure 1(b)).

### Fumitremorgin C alleviates AGE-induced SW1353 cell inflammation and ECM degradation

**Figure 2.**
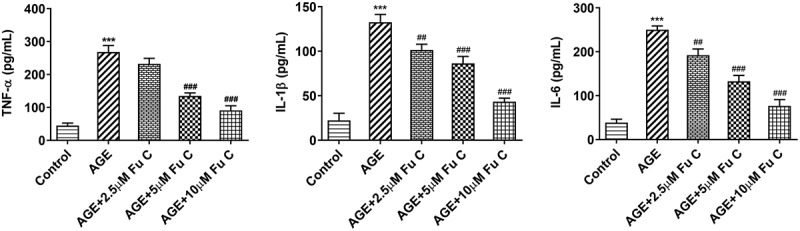
Fumitremorgin C alleviates AGE-induced SW1353 cell inflammation. The expression of TNF-α, IL-1β, and IL-6 in AGE-induced SW1353 cells pretreated with fumitremorgin C. ***P < 0.001 Versus Control. ^##^P < 0.01, ^###^P < 0.001 Versus AGE. Fu C: Fumitremorgin C.

**Figure 3.**
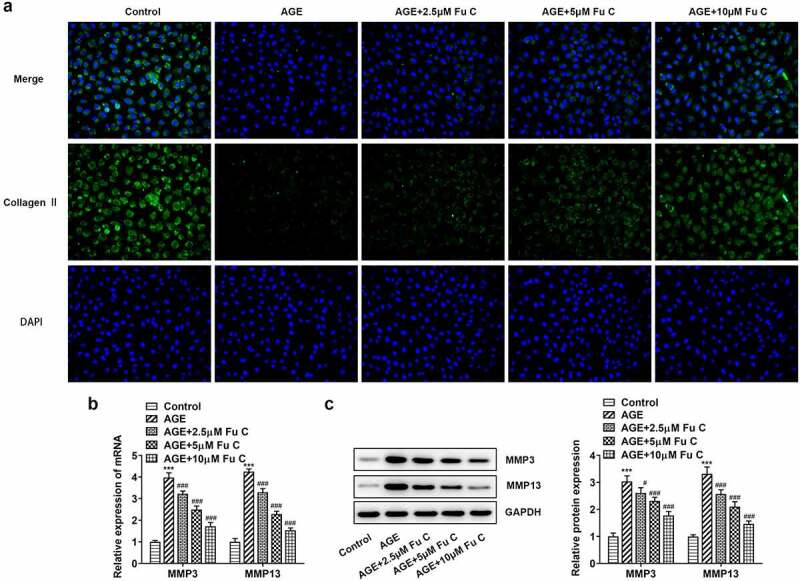
Fumitremorgin C alleviates ECM degradation in AGE-induced SW1353 cells. (a) The expression of collagen II and (b) ECM-related proteins in AGE-induced SW1353 cells pretreated with fumitremorgin C. ***P < 0.001 Versus Control. ^#^P < 0.05, ^###^P < 0.001 Versus AGE. Fu C: Fumitremorgin C.

**Figure 4.**
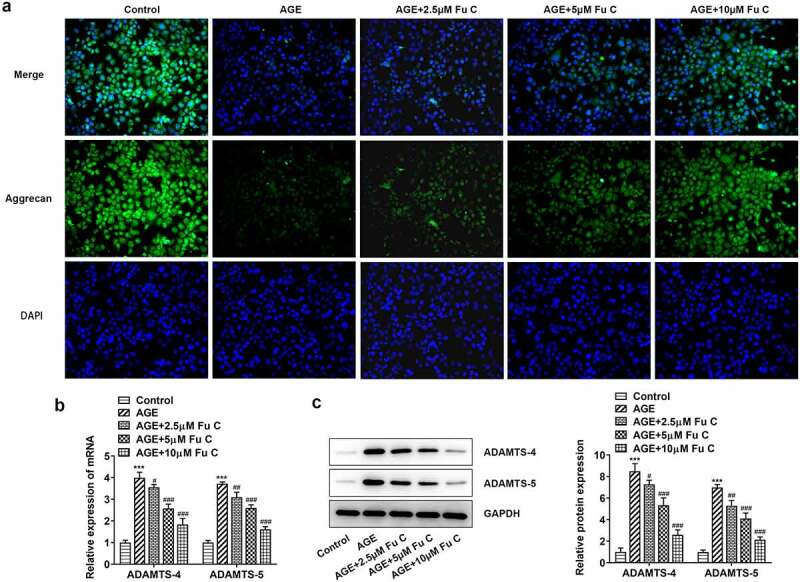
Fumitremorgin C alleviates ECM degradation in AGE-induced SW1353 cells. (a) The expression of Aggrecan and (b, c) ECM-related proteins in AGE-induced SW1353 cells pretreated with fumitremorgin C. ***P < 0.001 Versus Control. ^#^P < 0.05, ^###^P < 0.001 Versus AGE. Fu C: Fumitremorgin C.

Then, the effect of fumitremorgin C on the inflammation of AGE-induced SW1353 cells was determined. Results in Figure 2 by ELISA analysis indicated TNF-α, IL-1β, and IL-6 expression in SW1353 cells was dramatically increased by AGE compared with control group but suppressed by fumitremorgin C compared with AGE group. ECM is reported to be involved in the development of OA [15]. Thus, the expression of collagen II and Aggrecan was detected by immunofluorescence assay. Marked upregulation in the expression of collagen II by fumitremorgin C addition restored the inhibition of AGE on SW1353 cells (Figure 3(a), 4(a)). Together with ADAMTS-4 and ADAMTS-5, MMP3 and MMP13 which can regulate the expression of collagen II were elevated by AGE while decreased by varying concentrations of fumitremorgin C (Figure 3(b,c), Figure 4(b,c)). However, aggrecan expression, which can be regulated by ADAMTS-4 and ADAMTS-5, presented the opposite trend. Taken together, fumitremorgin C alleviates AGE-induced SW1353 cell inflammation and ECM degradation.

### Fumitremorgin C inhibits the expression of NF-KB/MAPK signal by activating SIRT1

**Figure 5.**
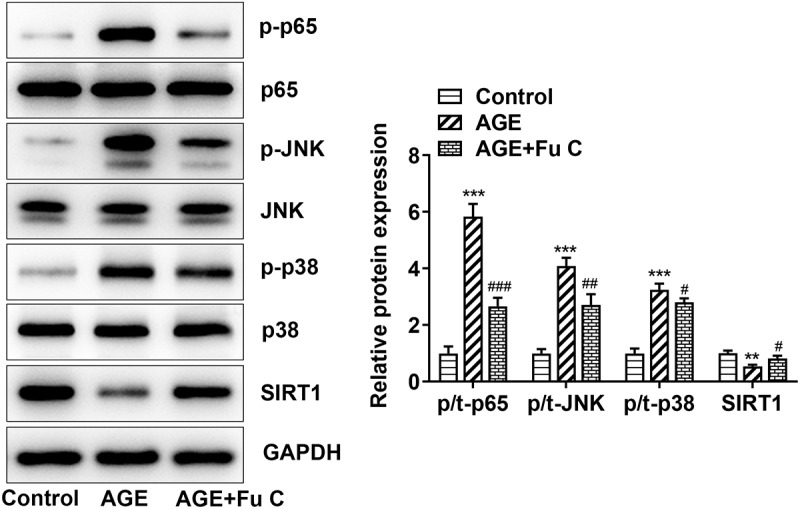
Fumitremorgin C inhibits the expression of NF-KB/MAPK signal by activating SIRT1. The proteins in NF-KB/MAPK signal were measured in AGE-induced SW1353 cells pretreated with fumitremorgin C. ***P < 0.001 Versus Control. ^#^P < 0.05, ^##^P < 0.05, ^###^P < 0.001 Versus AGE. Fu C: Fumitremorgin C.

In order to reveal the potential mechanism of fumitremorgin C, the expression of NF-KB/MAPK signal and SIRT1 were further examined. Obviously, the SIRT1 expression decreased by AGE when compared with control group was significantly recovered upon fumitremorgin C to SW1353 cells (Figure 5). 10 µM fumitremorgin C was used to pretreat cells for 2 h. Meanwhile, the protein levels of p-NF-KB p65, p-JNK, and p-p38 were increased by AGE as relative to control group and decreased by fumitremorgin C addition compared to AGE group. Thus, we can figure out that fumitremorgin C inhibits the expression of NF-KB/MAPK signal by activating SIRT1.

### SIRT1 inhibitor EX527 reverts the repressive effects of fumitremorgin C on AGE-induced SW1353 cell inflammation and ECM degradation

**Figure 6.**
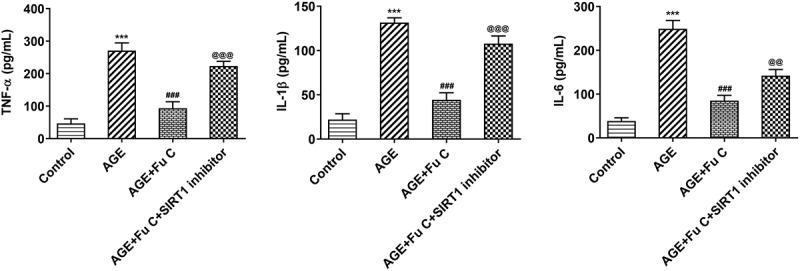
SIRT1 inhibitor EX527 reverts the repressive effects of fumitremorgin C on AGE-induced SW1353 cell inflammation. The expression of TNF-α, IL-1β, and IL-6 in AGE-induced SW1353 cells treated with fumitremorgin C and EX527. ***P < 0.001 Versus Control. ^###^P < 0.001 Versus AGE. ^@@^P < 0.01, ^@@@^P < 0.001 Versus + AGE+Fu C. Fu C: Fumitremorgin C.

**Figure 7.**
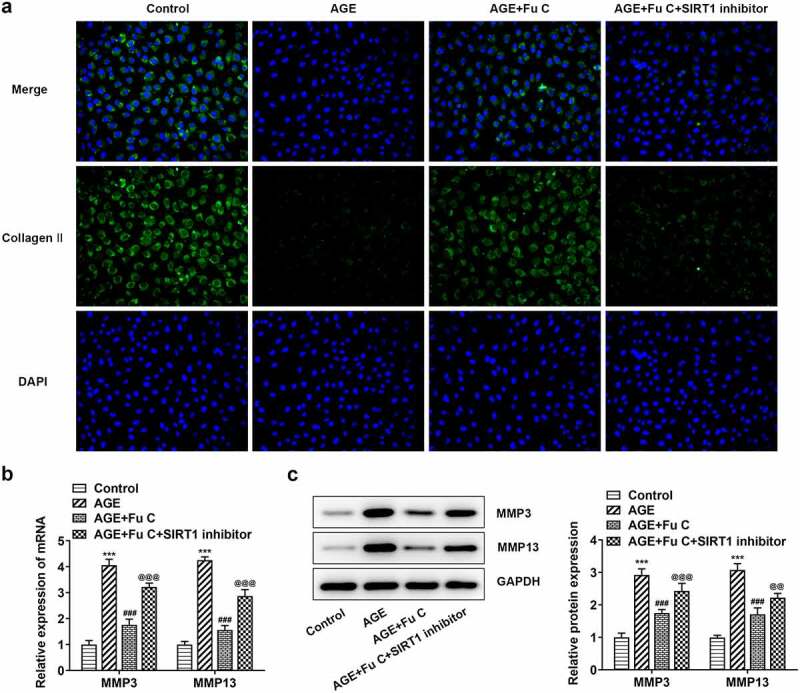
SIRT1 inhibitor EX527 reverts the repressive effects of fumitremorgin C on ECM degradation in AGE-induced SW1353 cells. (a) The expression of collagen II and (b, c) ECM-related proteins in AGE-induced SW1353 cells treated with fumitremorgin C and EX527. ***P < 0.001 Versus Control. ^###^P < 0.001 Versus AGE. ^@@^P < 0.01, ^@@@^P < 0.001 Versus + AGE +Fu C. Fu C: Fumitremorgin C.

**Figure 8.**
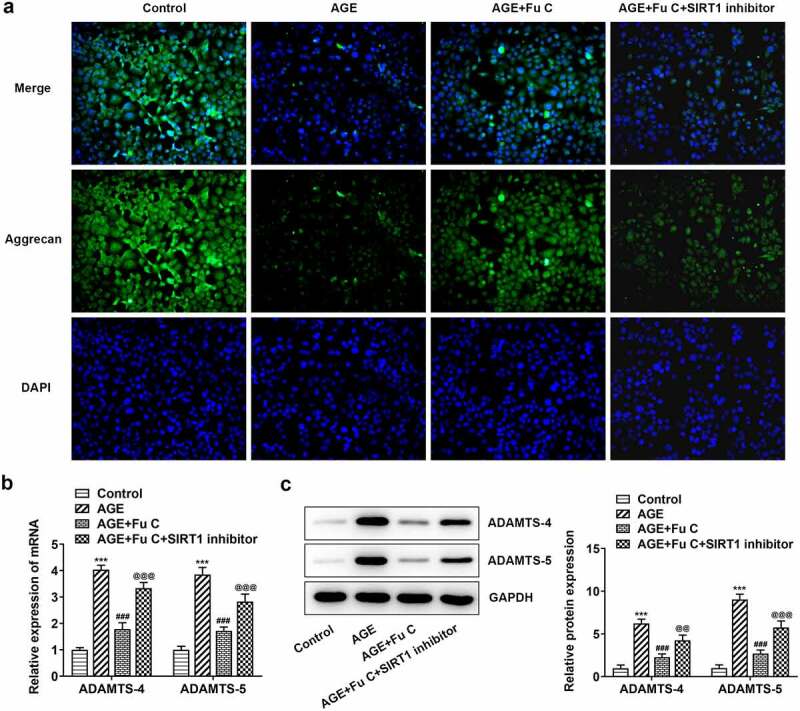
SIRT1 inhibitor EX527 reverts the repressive effects of fumitremorgin C on ECM degradation in AGE-induced SW1353 cells. (a) The expression of Aggrecan and (b, c) ECM-related proteins in AGE-induced SW1353 cells treated with fumitremorgin C and EX527. ***P < 0.001 Versus Control. ^###^P < 0.001 Versus AGE. ^@@@^P < 0.001 Versus + AGE+Fu C. Fu C: Fumitremorgin C.

To understand whether fumitremorgin C exerts repressive effects on AGE-induced SW1353 cell functions by regulation of SIRT1 expression, we pretreated SW1353 cells with 10 μm SIRT1 inhibitor EX527 for 2 h. We found that as compared to the AGE+10 μm group, the repressive effects of fumitremorgin C on the expression of TNF-α, IL-1β, and IL-6 were counteracted by EX527 pretreatment (Figure 6). As regards the effect of EX527 on the role of fumitremorgin C in ECM degradation, immunofluorescence showed that EX527 pretreatment inhibited expression of collagen II and Aggrecan in AGE-induced SW1353 cells exposed to fumitremorgin C (Figure 7(a), 8(a)). Moreover, Western blot analysis indicated that EX527 pretreatment resulted in MMP3, MMP13, ADAMTS-4 and ADAMTS-5 upregulation and aggrecan downregulation, as compared to AGE+10 μm group (Figure 7(b,c), Figure 8(b,c)).

## Discussion

Fumitremorgin C exerts numerous pharmacological properties such as reverting drug resistance to mitoxantrone, doxorubicin, and topotecan, which are drugs used for cancer therapies [16]. Currently, fumitremorgin C has been found to suppress the malignant progression of breast cancer [17]. An observation which aroused our research interest on the specific role of fumitremorgin C in bone-related disease such as OA was that it ameliorated osteoclastogenesis and bone resorption by mediation of RANKL-induced signaling pathways [13]. Fumitremorgin C alone did no cytotoxicity on the SW1353 cells, whereas AGE treatment induced notable damage in the cell viability. Concurrently, previous study also indicated that it restrained RANKL-induced osteoclastogenesis without obvious cytotoxicity on bone marrow macrophages in a concentration-dependent manner [13].

Epidemiologic statistics has linked systemic markers of inflammation to OA as increased levels of CRP and IL-6 were observed in the knee and joints of OA patients [18,19], and IL-6 and TNF-α have been particularly interpreted as predictors of the OA progression. In this study, the inflammation-related markers were upregulated as a result of AGE induction, whereas they were increasingly suppressed upon fumitremorgin C treatment. The proinflammatory state in OA patients would be a main reason for the pain from the joint tissues of the patients, and the word ‘inflamm-aging’ was coined to explain this status following the increase in patients’ age [20].

The response of the ECM in the articular cartilage, which is composed of a hydrated extracellular matrix (ECM) of collagens, proteoglycans and other proteins that embed a small amount of chondrocytes, to the mechanical stimulation is a significant factor in the progression of OA [21,22]. The sharply increased degradation of ECM as evidenced by reduced level of collagen II in AGE-induced SW1353 was restored by fumitremorgin C, indicating the repressive role of fumitremorgin C in ECM degradation thereby retarding the development of OA. The accumulation of AGEs in aging chondrocytes is related to dysregulated signaling pathways, and enhanced sensitivity to cytokines and chemokines, which activate the levels of MMPs and other inflammatory mediators [23,24]. The aggrecanases of the ADAMTS family of extracellular proteinases are the major aggrecan-degrading enzymes implicated in cartilage degradation in OA [25]. Herein, AGE as a stimulant triggered low expression of aggrecan and high expression of ADAMTS-4 and ADAMTS-5, which was reversed by fumitremorgin C treatment.

SIRT1 has the chondroprotective actions, and its activation can block cartilage degeneration due to aging or injury [26]. Correspondingly, we found that fumitremorgin C restored SIRT1 expression in the presence of AGE. It was reported that fumitremorgin C inhibited the activity of NF-κB remarkably [27]. The present work found thatthe NF-KB/MAPK was suppressed by fumitremorgin C via the activation of SIRT1. Specifically, the chondrocytes in OA shift to a degradative phenotype where the NF-KB transcription factors trigger the secretion of many degradative enzymes, including the matrix metallo-proteinases MMPs and the aggrecanases, ADAMTS4 and ADAMTS5, leading to articular cartilage breakdown [28]. It was reported that MAPK signaling pathway including p38 and JNK participates in cartilage degradation in an OA cell model [29]. Moreover, the addition of SIRT1 inhibitor EX527 reverted the inhibitory effects of fumitremorgin C on inflammation and ECM degradation, suggesting that fumitremorgin C contributes to an inhibitory effect on inflammation and ECM degradation via SIRT1 possibly through NF-KB/MAPK pathway.

## Conclusion

In conclusion, fumitremorgin C alleviates AGE-induced chondrocyte inflammation and collagen II and aggrecan degradation through SIRT1/NF-κB/MAPK, which reveals the underlying mechanism by which fumitremorgin C alleviates OA.

## Data Availability

The datasets used and/or analyzed during the current study are available from the corresponding author on reasonable request.

## Highlights

- Fumitremorgin C ameliorates inflammation and the degradation of CollagenII and aggrecan induced by AGEs.
- Fumitremorgin C modulates SIRT1/NF-κB/MAPK pathway to ameliorate inflammation.
- Fumitremorgin C suppresses the degradation of CollagenII and aggrecan by SIRT1/NF-κB/MAPK pathway.
